# Kinetics of mycolactone in human subcutaneous tissue during antibiotic therapy for *Mycobacterium ulcerans* disease

**DOI:** 10.1186/1471-2334-14-202

**Published:** 2014-04-15

**Authors:** Fred S Sarfo, Richard O Phillips, Jihui Zhang, Mohammed K Abass, Justice Abotsi, Yaw A Amoako, Yaw Adu-Sarkodie, Clive Robinson, Mark H Wansbrough-Jones

**Affiliations:** 1 Komfo Anokye Teaching Hospital, Kumasi, Ghana; 2 School of Medical Sciences, Kwame Nkrumah University of Science and Technology, Kumasi, Ghana; 3 St. George’s University of London, London, UK; 4 Agogo Presbyterian Government Hospital, Ministry of Health, Agogo, Ghana

**Keywords:** *Mycobacterium ulcerans*, Mycolactone, Biomarker, Antibiotic therapy, Prognosis, Treatment response

## Abstract

**Background:**

*Mycobacterium ulcerans* (*M. ulcerans*) causes a devastating necrotising infection of skin tissue leading to progressive ulceration. *M. ulcerans* is the only human pathogen that secretes mycolactone, a polyketide molecule with potent cytotoxic and immunomodulatory properties. These unique features make mycolactone an attractive biomarker for *M. ulcerans* disease. We sought to measure the concentration of mycolactone within lesions of patients with Buruli ulcer before, during and after antibiotic treatment to evaluate its association with the clinical and bacteriological response to therapy.

**Methods:**

Biopsies of *M. ulcerans* infected skin lesions were obtained from patients before, during and after antibiotic therapy. Lipids were extracted from the biopsies and concentration of mycolactone was assayed by mass spectrometry and a cytotoxicity assay and correlated with clinical and bacteriological response to therapy.

**Results:**

Baseline concentration of mycolactone measured by mass spectrometry predicted time to complete healing of small nodules and ulcers. Even though intra-lesional concentrations of mycolactone declined with antibiotic treatment, the toxin was still present after antibiotic treatment for 6 weeks and also 4 weeks after the end of treatment for 8 weeks in a subgroup of patients with slowly healing lesions. Additionally viable bacilli were detected in a proportion of these slowly healing lesions during and after treatment.

**Conclusions:**

Our findings indicate that baseline intra-lesional mycolactone concentration and its kinetics with antibiotic therapy are important prognostic determinants of clinical and bacteriological response to antibiotic treatment for *Mycobacterium ulcerans* disease. Mycolactone may be a useful biomarker with potential utility in optimising antibiotic therapy.

## Background

*Mycobacterium ulcerans* infection causes Buruli ulcer, a chronic necrotising skin infection with high prevalence in rural West Africa. *M. ulcerans* infection may manifest initially as a pre-ulcerative nodule, a plaque or as a rapidly progressing oedema which breaks down to form characteristic ulcers with undermined edges. The virulence of *M. ulcerans* is dependent on mycolactone, a lipid toxin with cytotoxic or immunosuppressive properties depending on its concentration [1-6]. Histopathology of Buruli ulcer shows clumps of extracellular bacteria surrounded by necrotic subcutaneous fat with little inflammation around the organisms. Mycolactone, which is responsible for the necrosis, is synthesised by giant polyketide synthases and polyketide modifying enzymes whose genes are carried on a 174-kb plasmid known as pMUM001 [7]. The resulting molecule consists of a 12-membered lactone ring with two polyketide derived side chains. Variation in the structure of polyketide side chains is responsible for differences in virulence/potency among the family of mycolactones.

Mycolactone causes a cytopathic effect on mouse fibroblasts [8], adipocytes [9], primary human keratinocytes [10] and human embryonic lung fibroblasts [11]. At lower concentrations *in-vivo* mycolactone suppresses dendritic cell, macrophage and T-cell function abrogating cytokine and chemokine secretion in response to mitogens [4], an effect which has been shown to be mediated post-transcriptionally [12]. It also impairs T-cell homing by suppressing microRNA let-7b control of L-selectin (CD62-L) expression [13].

We have detected mycolactone associated cytotoxicity in lipid extracts from *M. ulcerans* infected human subcutaneous tissue and showed that mycolactone was present by mass spectrometry [11]. *M. ulcerans* is the only human pathogen known to be associated with mycolactone secretion and therefore it may be a useful biomarker. For instance, profound differences have been demonstrated in time to healing of similar-sized Buruli ulcers on standard antibiotic therapy with daily oral rifampin and intramuscular streptomycin for 8 weeks [14] and persistence of mycolactone in wounds may contribute to this observation. In a murine model, we have recently demonstrated that the decline in tissue mycolactone concentration during antibiotic therapy tracked closely with reduction in colony forming units of *M. ulcerans* and with resolution of footpad swelling, highlighting the close association between mycolactone and pathogenesis of *M. ulcerans* disease [15]. In the present studies we have investigated the dynamics of clearance of mycolactone from skin tissues during curative antibiotic treatment in order to provide proof-of-principle data on the prognostic potential of mycolactone as a biomarker.

## Methods

### Ethical statement

The study protocol was approved by the ethics review committee at the School of Medical Sciences, Kwame Nkrumah University of Science and Technology, Kumasi, Ghana. All patients provided written informed consent before inclusion into the study. For minors informed consent was obtained from parents or guardians on their behalf.

### Subjects and samples

Eighty (80) patients with PCR confirmed diagnosis of *M. ulcerans* disease recruited from villages around the Ahafo Ano North, Ashanti Akim North and Nkawie districts at the Tepa Government Hospital, the Agogo Presbyterian Hospital and the Nkawie Government Hospital of the Ministry of Health, Ghana were selected for this study. After the patients had given informed consent, a fine needle aspiration and two 4 mm punch biopsies were obtained from the centre of non-ulcerated lesions or from the edge of the viable skin around ulcers after application of a topical anaesthetic for 15 minutes. The fine needle aspirate was used to confirm the diagnosis by PCR. One punch biopsy was used to perform microscopy and quantitative culture and the other biopsy was placed in amber-coloured tubes, snap frozen in liquid nitrogen and stored at -70°C for lipid extraction to detect and quantify mycolactone. Patients were treated with antibiotics for 8 weeks, either using the combination of rifampin and streptomycin throughout (RS8) or substituting rifampin plus clarithromycin after 2 weeks (RS2RC6). In consenting adults two further biopsies were obtained, one for quantitative culture and one for lipid extraction and mycolactone quantification at 6 weeks during antibiotic therapy and, from lesions which had not healed, at 12 weeks.

### Diagnostic confirmation of *M. ulcerans* disease in study subjects

PCR was performed on fine needle aspirates from infected tissue targeting the *IS2404* insertion sequence of *M. ulcerans* as described elsewhere [16]. The PCR was based on the *IS2404* insertion element; PU4F (5′-GCGCAGATCAACTTCGCGGT-3′) and PU7Rbio (5′-GCCCGATTGGTGCTCGGTCA-3′) were used as primers. Microscopic examination to detect *M. ulcerans* was performed by homogenising skin biopsy tissue obtained by punch biopsy and staining by the Ziehl-Neelsen technique. For quantitative culture, 1 ml of homogenized tissue was decontaminated by the modified Petroff method for 10 minutes after which four 10-fold serial dilutions were prepared and 100 μl of homogenate at each concentration was inoculated in triplicate onto Löwenstein-Jensen slopes. The remaining tissue homogenate at each dilution was also inoculated and cultures were incubated at 31°C and examined weekly for 6 months before they were discarded. Positive cultures were tested for *IS2404* to confirm the identity and presence of *M. ulcerans*.

### Monitoring the clinical response to antibiotic treatment

The maximum diameter of the lesion and the diameter at right angles were measured and the average diameter was used. Lesions were categorised as category I when the average diameter was less than 5 cm, category II between 5 to 15 cm and category III more than 15 cm in diameter or multiple lesions. The clinical response to antibiotic treatment was monitored every two weeks to establish the time to complete healing and monthly thereafter for one year from completion of antibiotic treatment to check for recurrences. For ulcerative lesions, time to healing was defined as time to complete re-epithelialization or stable scab formation whereas for nodular lesions, time to healing was defined as time to complete resolution such that no visible or palpable lesion remained.

### Extraction of lipids from human skin for mycolactone detection and quantification

Lipids were extracted from skin biopsies from Buruli lesions using chloroform: methanol 2:1 (vol/vol) followed by a Folch extraction with 0.2 volumes water as previously described [11]. Briefly, the punch biopsy was weighed and placed in a 1.5 ml green top matrix tube containing 500 μl of diatoms (Q-bio) and homogenised in extraction solution for 45 seconds at a power of 6.5 in a Fast Prep Ribolyser. The homogenate was transferred into a microfuge tube and allowed to stand for 30 minutes. After centrifugation at 10,000 g for 15 minutes the organic phase was harvested. The organic phase was dried in a roto-evaporator and re-suspended in ice-cold acetone for 1 hour to precipitate phospholipids. Acetone soluble lipids (ASL) suspended in 100 μl ethanol for mycolactone detection by mass spectrometry and cytotoxicity assays were kept in amber-coloured Eppendorf tubes at -80°C until analyses were performed. To determine the efficiency of the extraction technique, 4 mm punch biopsies from an excised human breast tissue (obtained with permission from Department of Surgery) were spiked with 1ug of synthetic mycolactone A/B and subjected to extraction as described above.

### Quantitative cytotoxicity assay

Mycolactone present in ASL from tissues were quantified using the MTT assay, a colorimetric test that measures cellular metabolic activity of human embryonic lung fibroblasts (HELF) [11,15,17] as previously described. Briefly HELF cells (kindly donated by Dr. Kay Capaldi) were maintained in Dulbecco’s modified Eagle’s medium supplemented with 10% foetal calf serum and 2 mM L-glutamine in the presence of penicillin and streptomycin 100 mU/ml and 100 mg/ml respectively and incubated in 5% carbon dioxide at 37°C. For cytotoxicity assays proliferating HELF cells were seeded at a density of 10^5^/well in microtitration plates overnight.

ASL from lesions, positive controls (purified mycolactone, synthetic mycolactone and mycolactone spiked human skin tissue) and negative controls (lipids from an excised breast tissue obtained at mastectomy) were dissolved in 100 μl of absolute ethanol and 2-fold dilutions performed up to the 5th dilution. 5 μl of each dilution was used to treat HELF cells in triplicate. After incubation, 20 μl of 5 mg/ml MTT (Sigma) was added to each well and incubated for a further 4 h for purple coloured formazan crystals to develop following which 100 μl isopropanol: HCl (2 N) was used in a ratio of 49:1 to dissolve formazan crystals for spectrophotometric quantification in a multiplate well reader at 570-690 nm. Time course and dose-response calibration curves of mycolactone mediated cytotoxicity on HELF cells were performed by treating HELF cells over 24, 48 and 72 hours with serial dilutions of purified or synthetic mycolactone A/B (kind gifts of Dr. Caroline Demangel, Pasteur Institute, Paris, France and Professor Y Kishi, Harvard University, USA respectively) up to concentration of 250 ng/ml following which curve-fitting plots were constructed. In order to evaluate non-specific cytotoxicity caused by other skin lipids, lipid extracts from non-infected human skin were tested for cytotoxicity and this was set as the lower limit of detection for the cytotoxicity assay. All calibration and quantification experiments were performed three times.

### Characterization and quantification of mycolactone A/B by UPLC tandem mass spectrometry

Liquid chromatographic separation and tandem mass spectrometric detection were performed using an ABI Sciex 3200 Q Trap mass spectrometer (ABI Sciex, UK) interfaced with a Shimadzu UFLC system (UFLC XR system with CBM-20A controller, SIL-20 AC XR Prominence autosampler, CTO-20 AC Prominence column oven and LC-20 AD XR pumps (Shimadzu, UK). For the analysis of lipid extracts from clinical specimens, positive and negative controls, UPLC was combined with enhanced product ion identification (LC-EPI) or multiple reaction monitoring (LC-MRM) as previously described (15).

### Statistical analysis

Medians were compared using the Mann-Whitney’s *U*-test for two independent continuous variables. Correlations between two independent variables were analyzed using Pearson’s correlation test. GraphPad Prism version 4 software was used for all analysis with p < 0.05 considered statistically significant.

## Results

### Demographics, diagnostic and clinical data of study participants

Eighty patients with clinically confirmed *M. ulcerans* disease were included with median age of 14 years (range 5 – 70 years) and male to female ratio 1:1. There were 18 (22.5%) nodules, 14 (17.5%) plaques, 44 (55.0%) ulcers and 4 (5.0%) oedematous lesions (Table 1). There were 50 (62.5%) category I lesions, 23 (28.8%) category II lesions and 7 (8.7%) category III lesions. The median reported duration of lesions before presentation was 3 weeks (range 1–32 weeks) with no significant differences between the clinical forms of *M. ulcerans* disease. PCR for *IS2404* was positive in all patients; 40 out of 60 cultures for *M. ulcerans* (66%) were positive and acid-fast bacilli were positive in 25 out of 60 (42%) samples tested before antibiotic therapy. The median time to healing of lesions in patients treated with RS2RC6 was 16 weeks (interquartile range (IQR) 4–36) [18]. Among patients who received rifampin and streptomycin for 8 weeks, the median time to healing was 14 weeks (IQR 4-48) which was not significantly different.

**Table 1:** Demographic, diagnostic and clinical data of study participants

**Characteristic**	**Form of *M. ulcerans *** **lesion**				
	**Nodule n = 18**	**Plaque n = 14**	**Oedema n = 4**	**Ulcer n = 44**	**Total n = 80**
Male: Female	12:6	5:9	2:2	22:22	41:39
Median (range) age	13.5 (5-48)	12 (5-70)	16 (7-55)	16 (5-59)	14 (5-70)
Lesion category					
Category 1(< 5 cm)	18	10	0	22	50
Category 2 (5-15 cm)	0	4	2	17	23
Category 3 (> 15 cm)	0	0	2	5	7
Median (range) duration of lesion (weeks)	4 (1 – 12)	3 (1-12)	3.5 (3-4)	3 (1 – 32)	3 (1 – 32)
PCR result: no. of positive tests	18	14	4	44	80
Culture result (+: - : nd)	8:6:4	8:5:1	1:1:2	23:8:13	40:20:20
Microscopy result (+: - : nd)	7:7:4	6:7:1	0:2:2	12:19:13	25:35:20
Median (range) time to healing (weeks)	8 (4–32)^1^	31 (2–48)^2^	8 (6-12)^3^	13 (5-44)^4^	12 (2-48)
Recurrence 1 year after treatment	0	0	0	0	0

nd = not done.

^1^Excluding one patient lost to follow up.

^2^Excluding one patient whose lesion had not healed after 52 weeks and required surgery.

^3^Excluding two patients with extensive oedematous disease who required skin grafting.

^4^Excluding four patients lost to follow up and 2 ulcers that required skin grafting.

### Experimental data on assays for detection and quantification of mycolactone

#### ***Cytotoxicity assay***

Mycolactone mediated cytotoxicity on human embryonic lung fibroblasts followed a sigmoidal dose response at 24, 48 and 72 h (see Additional file 1: Figure S1A) and incubation for 48 hours resulted in optimal best-fit plots (r^2^ = 0.98) even though inter-assay variations were observed in the quantities of mycolactone measured by the cytotoxicity assay (see Additional file 1: Figure S1B). Synthetic mycolactone induced 50% cytotoxicity at a concentration of 4.7 ng/ml. The lower limit of detection was set at 20% cytotoxicity to exclude non-specific cytotoxicity. This lower limit was set by measuring the cytotoxicity induced by lipid extracts from uninfected skin tissue used as negative control.

#### ***Mass spectrometry***

**Figure 1:**
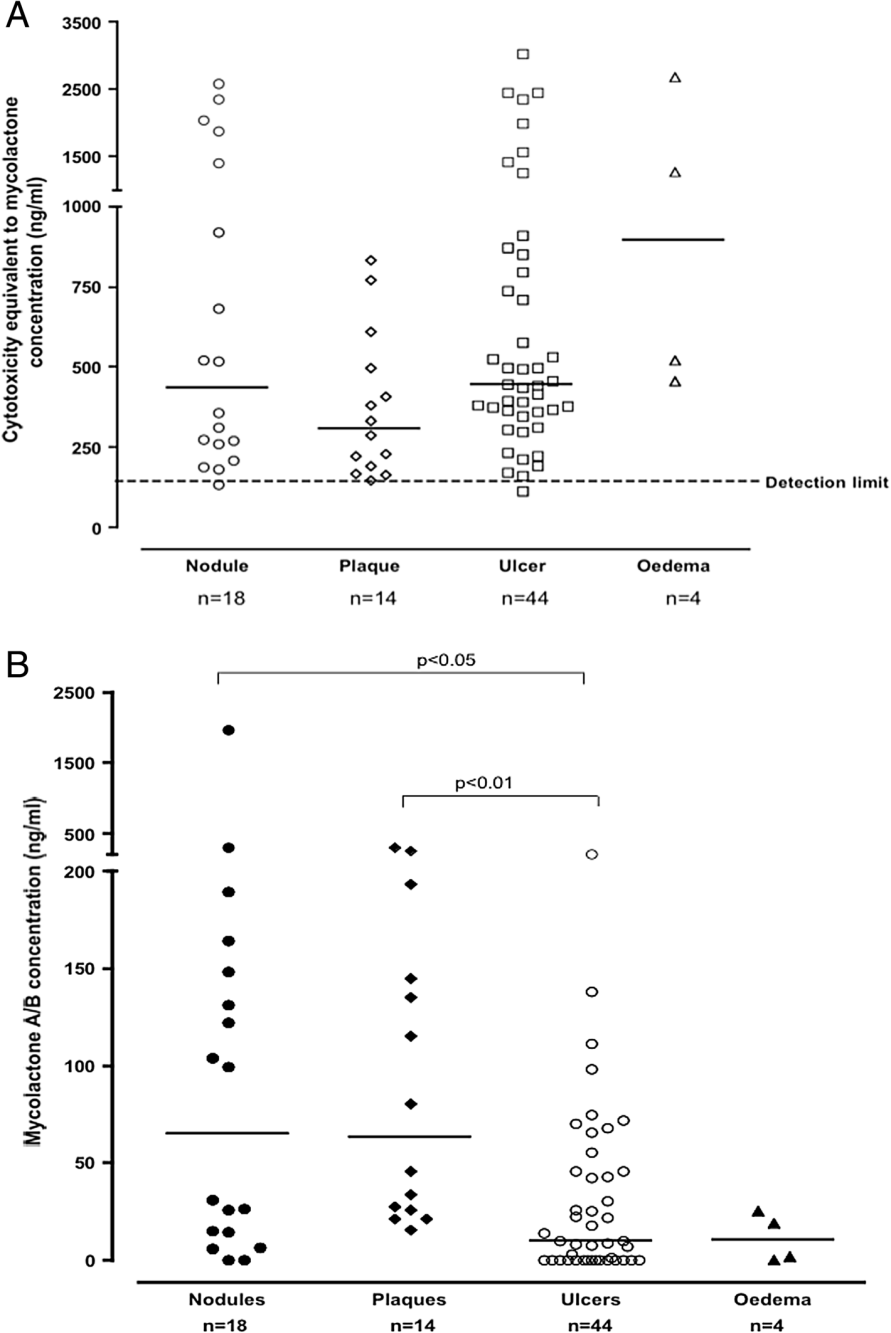
**Mycolactone concentration in punch biopsies of untreated *M. ulcerans *disease lesions before antibiotic treatment. (A)** Mycolactone concentration in ASL measured by cytotoxicity assay using synthetic mycolactone A/B generated calibration curves. Horizontal lines represent medians and each dot represents one lesion. **(B)** Mycolactone concentration in punch biopsies of untreated *M. ulcerans* disease lesions before antibiotic treatment. Mycolactone A/B concentration in ASL measured by multiple reaction monitoring (MRM) methodology with liquid chromatographic separation coupled to a tandem mass spectrometer. Horizontal lines represent medians and each dot represents one lesion.

Mycolactone detected in skin extracts showed the same mass spectra as those of synthetic mycolactone A/B and also contained a number of other mycolactone associated ions (e.g. m/z 659.6 and 747.7) (see Additional file 1: Figure S2). Figure 1 in Additional file 1 also shows extracted ion chromatograms (XIC) of the m/z 429.6 ion selected for quantification of mycolactone by LC-MRM analysis. EPI mass spectra indicated a propensity of authentic mycolactone A/B to form a sodium adduct (m/z 765.7, (M + Na^+^), (see Additional file 1: Figure S2). Fragment ions at m/z 429.6 and 359.5 correspond to the core lactone and polyketide side chains respectively. Mycolactone A/B detected in skin extracts displayed similar mass spectra and also contained a number of other ions (e.g. m/z 659.6 and 747.7) that were present in authentic reference material. m/z 747.7 is derived from m/z 765.7 with loss of water and generates m/z 659.6 with loss of C_4_H_9_O_2_. The lower limit of detection of mycolactone A/B by mass spectrometry was 10 pg.

#### ***Recovery of mycolactone from spiked skin tissue***

The recovery of synthetic mycolactone A/B from spiked skin tissue was 11-18% for a 1 μg spiked skin sample (n = 3 independent experiments) using mass spectroscopy compared with a recovery of 64-100% (n = 3 independent experiments) using the cytotoxicity based method of quantification. Mycolactone concentration from lipid extracts from each 4 mm skin biopsy was expressed as ng/ml.

### Mycolactone detection and concentration in *M. ulcerans* infected human skin lesions

#### ***In untreated nodules, plaques, ulcers and oedematous lesions***

Compared with the conventional PCR for *IS2404*, the sensitivity of mycolactone detection was 92% by cytotoxicity and 77% by mass spectrometry. The median (range) concentration of mycolactone in 80 untreated lesions measured by mass spectrometry was 26 ng/ml (0-1970), significantly lower than that measured by cytotoxicity at 439 ng/ml (136–3020) (p < 0.0001). Figure 1 shows the concentration of mycolactone in four forms of Buruli lesions before antibiotic treatment. There was a wide variation of concentration in all types of lesion with median (range) of 437 ng/ml (136–2589; n = 18) in nodules, 311 ng/ml (148-834; n = 14) in plaques, 443 ng/ml (114–3020; n = 44) in ulcers and 895 ng/ml (457-2689; n = 4) in oedematous lesions using the cytotoxicity assay (Figure 1A). Using mass spectrometry, the median concentration in nodules and plaques was significantly higher than that in ulcers (Figure 1B) whereas there was no significant difference by cytotoxicity (Figure 1A). Cytotoxicity was high among the four oedematous lesions but the amounts of intact mycolactone detected by mass spectrometry were low.

#### ***Distribution of mycolactone within infected tissues***

Mycolactone concentration was measured by cytotoxicity in paired biopsies taken from the centre and the periphery of six pre-ulcerative lesions (3 nodules and 3 plaques). There was a trend towards a centripetal gradient in 5 out of 6 lesions. The median (range) concentration of mycolactone by mass spectrometry at the centre was 110 ng/ml (18-285), significantly higher than 26 ng/ml (0-75) at the periphery (p < 0.05).

#### ***Mycolactone concentration during and after antibiotic therapy***

**Figure 2:**
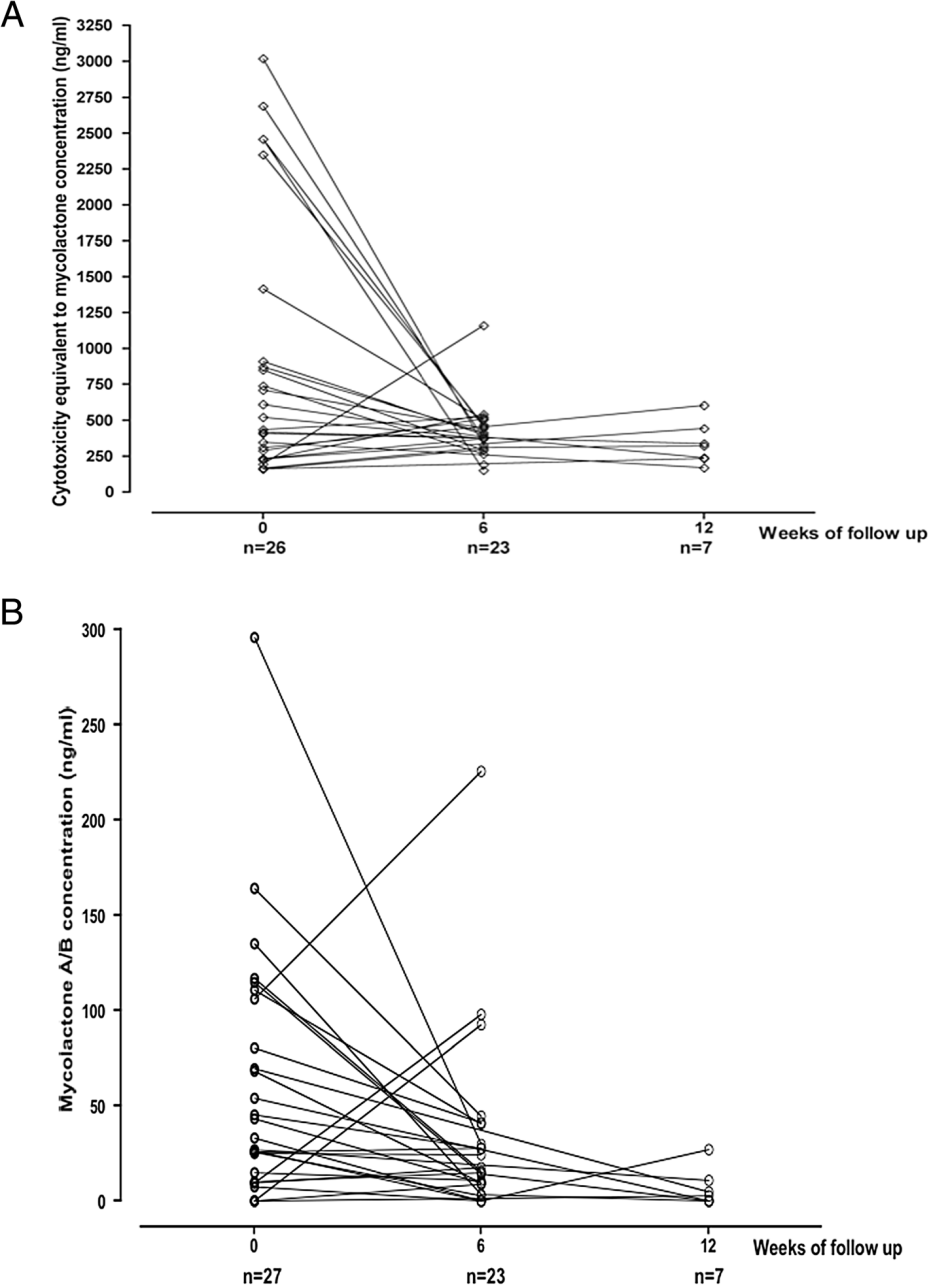
**Tissue mycolactone concentration in serial biopsies during and after antibiotic treatment.** Antibiotic treatment was started at week 0 and completed at week 8. Biopsies were taken at weeks 0 in all patients and at week 6 and/or 12 when it was clinically indicated. **(A)** Mycolactone concentration in ASL measured by cytotoxicity assay using synthetic mycolactone A/B generated calibration curves. **(B)** Tissue mycolactone concentration in serial biopsies during and after antibiotic treatment. Mycolactone A/B concentration in ASL measured by multiple reaction monitoring (MRM) methodology with liquid chromatographic separation coupled to a tandem mass spectrometer.

Further biopsies were taken at 6 and/or 12 weeks from patients whose lesions were healing slowly. Median concentration of mycolactone by cytotoxicity decreased from 479 ng/ml (range 159-3020; n = 26) at week 0 to 385 ng/ml (153-1160, n = 23) (p < 0.05) at 6 weeks and to 320 ng/ml (169-604; n = 7) (NS) at 12 weeks (Figure 2A). By mass spectrometry, the median concentration decreased from 34 ng/ml (range 0–296) at week 0 to 16 ng/ml (0-226) (p < 0.05) at week 6 and to 3 ng/ml (0-27) (NS) at week 12 (Figure 2B).

#### ***Correlation between healing time and mycolactone concentration at baseline***

**Figure 3:**
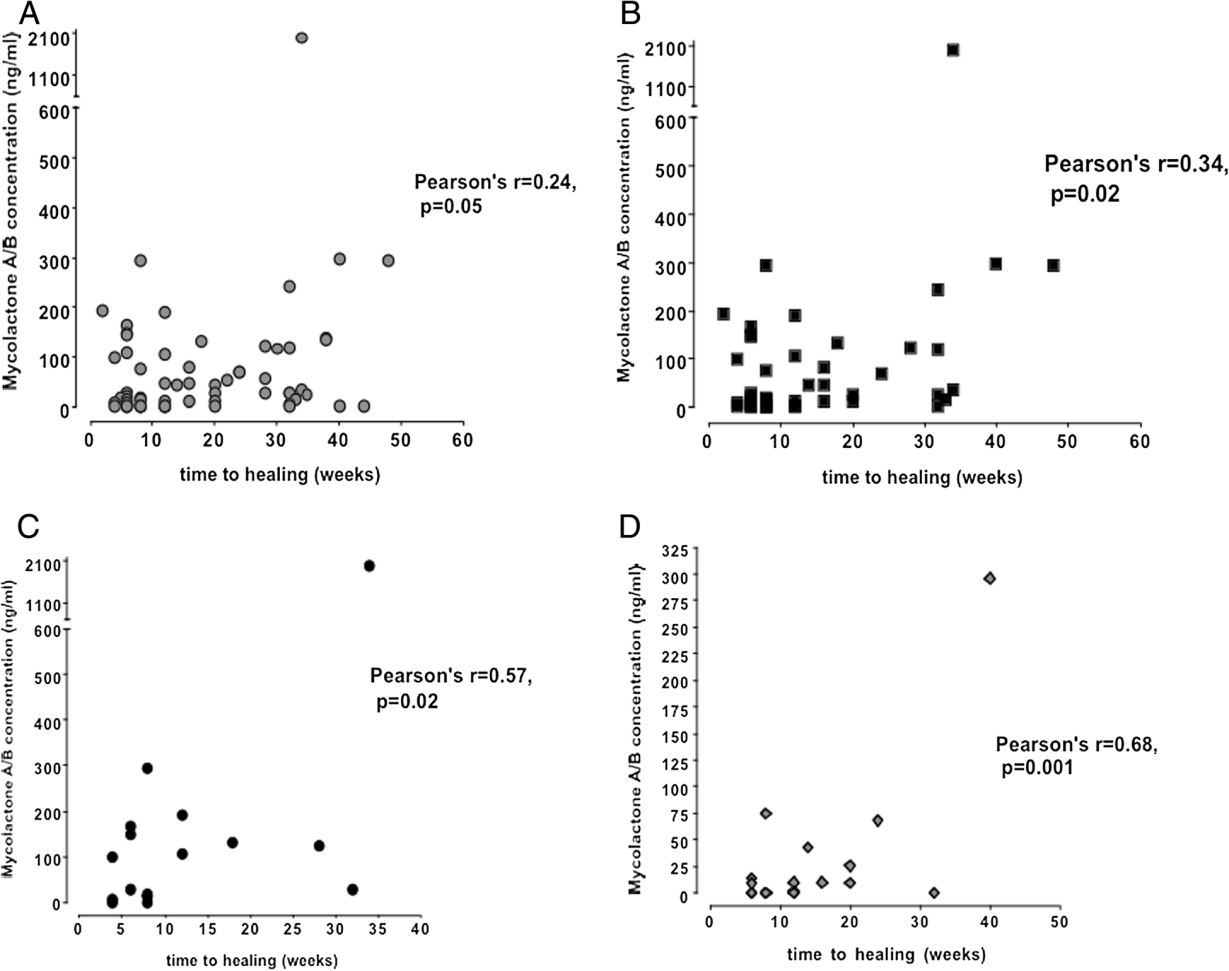
**Correlation between baseline mycolactone A/B concentration measured using mass spectrometry and time to complete healing. (A)** All categories and forms of buruli lesion. **(B)** All category I lesions. **(C)** Nodular lesions. **(D)** Category I ulcers (less than 5 cm in widest diameter).

There was a weak but significant correlation between mycolactone measured by mass spectrometry at baseline with time to complete healing (Pearson’s correlation coefficient 0.24; p = 0.05). A stronger correlation between time to healing and category I lesions (r = 0.34, p = 0.02), among nodular lesions (r = 0.57, p = 0.02) and category I ulcerative lesions (r = 0.68, p = 0.001) in particular (Figure 3A-D) were noted. Nodules with mycolactone concentration below 64.9 ng/ml (the median value) healed in 8 weeks (range 4-32) compared with 12 weeks (6-52) for those with mycolactone concentration above the median (p < 0.05).

### Mycolactone detection in lesions sampled for *M. ulcerans* culture

Samples were taken for culture of *M. ulcerans* from 60 patients before antibiotic treatment, 15 patients at week 6 during antibiotic therapy and 3 patients at week 12 (i.e. 4 weeks after completing antibiotic treatment). The results of culture and mycolactone detection at weeks 0, 6 and 12 are shown in Table 2. Before antibiotic therapy, 66% of PCR positive lesions were also culture positive whilst cytotoxicity was detected in 87% of lesions and mycolactone was detected by mass spectrometry in 70% of lesions in this subset of 60 patients. Despite higher overall sensitivity, mycolactone was not detected in some samples that were culture positive at week 0; 5 by cytotoxicity and 14 by mass spectrometry. At week 6, mycolactone was present in 7 culture negative samples using both the cytotoxicity assay and mass spectrometry. At week 12, mycolactone was detected by cytotoxicity in 2 culture positive lesions but mass spectrometry was negative.

**Table 2:** **Mycolactone detection by mass spectrometry and cytotoxicity assay in patients with *M. ulcerans *** **disease sampled for *M. ulcerans *** **culture**

**Time**	**Week 0**		**Week 6**		**Week 12**	
**Mycolactone detection**	**Culture result**		**Culture result**		**Culture result**	
	**Positive**	**Negative**	**Positive**	**Negative**	**Positive**	**Negative**
Cytotoxicity positive	35	17	7	7	2	0
Cytotoxicity negative	5	3	0	1	0	1
Total (1)	**40**	**20**	**7**	**8**	**2**	**1**
MS positive	26	16	6	7	0	0
MS negative	14	4	0	1	2	1
Total (2)	**40**	**20**	**6**	**8**	**2**	**1**

MS - Mass spectrometry. Total (1) represents sub-total of comparison of mycolactone detection by cytotoxicity and culture results. Total (2) represents sub-total of comparison of mycolactone detection by mass spectrometry and culture results.

## Discussion

Our study has highlighted the potential importance of mycolactone detection and quantification in infected human subcutaneous tissue as a tool in understanding the pathogenesis of Buruli ulcer disease and in refining antibiotic treatment. Using mass spectrometry, significantly higher concentrations of mycolactone were detected in nodules and plaques, the early forms of *M. ulcerans* disease, than in ulcers. There was considerable variation in mycolactone concentration in these lesions (Figure 1A and 1B) which may reflect differences in the duration of infection and bacterial burden at presentation; if the patient had been infected recently, the lesions would be expected to contain a lower load of bacilli secreting mycolactone. There was some correlation between concentration of mycolactone A/B and healing time in this sub-group and this could be an important observation for planning treatment.

In the small number of oedematous lesions studied, there was a low concentration of mycolactone A/B by mass spectrometry but high cytotoxicity. This is the least common but most aggressive form of early *M. ulcerans* disease in which oedema spreads rapidly to adjacent areas leading to formation of large ulcers. The pathogenesis of the oedema is unknown but the discrepancy between mycolactone concentration by mass spectrometry and the cytotoxicity assay suggests that either there was a high turnover of mycolactone or that an anomalous host response to *M. ulcerans* infection generated cytotoxic lipid molecules of a different sort. This needs further investigation.

The centripetal gradient of mycolactone concentration from high in the centre to low in the periphery of pre-ulcerative lesions is compatible with the clinical observation that lesions tend to break down in the centre to form ulcers but variations in mycolactone concentration have to be interpreted with caution since only a small number of samples could be taken for ethical reasons. Sampling error is an unavoidable problem in studies of mycolactone concentration in human tissue but we have tried to limit it by studying a large number of patients.

Mycolactone associated cytotoxicity was still positive in some biopsies obtained 6 weeks into antibiotic therapy and 4 weeks after its completion from a subset of slowly healing lesions and in some cases *M. ulcerans* cultures were also positive (Table 2). Mycolactone A/B was detectable in most of the 6 week samples but not at 12 weeks. This may indicate that a proportion of patients with slowly healing lesions still had viable bacteria in the skin late in the course of treatment and some of these lesions also had positive cultures for *M. ulcerans.* It is also possible that mycolactone persisted in lesions after the bacteria were killed but the fact that we also found some positive cultures after antibiotic treatment argues against this. To resolve this question further work will be needed using new techniques for detecting viable *M. ulcerans* such as the combined 16S rRNA and IS2404 assay [19].

In an earlier study, cultures of excised nodules and plaques from patients with early Buruli ulcer disease treated with streptomycin and rifampicin for periods varying from 2 to 8 weeks were culture negative from 4 weeks onwards [20]. This difference may be explained by the fact that patients with all forms of the disease were included in the present study. There was no difference between the RS8 and RS2RC6 groups with regard to the number of samples positive for mycolactone or bacterial culture at 6 or 12 weeks and the median time to healing was similar so it is unlikely that slow healing was due to reduced exposure to streptomycin [21].

Clinical observations have shown that some small Buruli lesions take a considerable time to heal with antibiotic therapy [14,22] and our study has provided some evidence to explain these observations. The persistence of mycolactone within Buruli lesions during and after antibiotic therapy could retard healing by killing keratinocytes [23] and by inhibiting the secretion of growth factors required for wound healing [24]. At the molecular level, mycolactone has recently been shown to bind to the Wiskott-Aldrich syndrome protein (WASp) in epithelial cells, provoking uncontrolled activation of Arp2/3. This disrupts assembly of actin in the cytoplasm resulting in defective cell adhesion which could lead to defective wound closure [25]. Compounds that inactivate mycolactone might therefore play an adjunctive role in promoting healing of Buruli ulcers.

Detection of viable organisms may be an important tool for determining the optimal duration of antibiotic treatment and the present study suggests that mycolactone measurement may have a role in this. Early lesions containing low numbers of viable organisms may be cured by antibiotic treatment for less than the standard 8 weeks and, on the other hand, ulcers still containing mycolactone at 6 weeks may require more prolonged treatment.

## Conclusions

We have shown that the baseline concentrations of tissue mycolactone as well as its kinetics during and after antibiotic therapy are important determinants of clinical response to treatment. Significantly, slowly healing lesions which were found to contain mycolactone also frequently had viable bacilli late into the recommended 8-week course of antibiotic therapy. In conclusion the measurement of tissue mycolactone is a promising new method of monitoring the clinical response to antibiotic therapy which may lead to new insights into the pathogenesis of *M. ulcerans* disease as well as guiding future treatment developments.

## Abbreviations

ASL: Acetone soluble lipids; PCR: Polymerase Chain Reaction; IS: Insertion sequence; MTT: 3-[4,5-dimethylthiazol-2-yl]-2,5 diphenyl tetrazolium bromide; UPLC: Ultra-performance liquid chromatography; UFLC: Ultra-fast liquid chromatography; EPI: Enhanced Product Identification; MRM: Multiple Reaction Monitoring.

## Competing interests

The authors declare that they have no competing interest.

## Authors’ contributions

FSS, ROP, YAS, MWJ coordinated and supervised the research. FSS, ROP, MWJ designed the study. FSS, ROP, MAK, JA, YAA coordinated the recruitment and management of patients. JZ, CR performed the measurement of mycolactone using mass spectrometry. FSS conducted cell culture experiments and statistical analysis. FSS and MWJ wrote the first draft of the manuscript. All authors revised the manuscript and contributed to improving the paper. All authors read and approved the final manuscript.

## Pre-publication history

The pre-publication history for this paper can be accessed here:

http://www.biomedcentral.com/1471-2334/14/202/prepub

## Supplementary Material

Additional file 1 **Experimental data on mycolactone quantification assays. Figure S1A and 1B** shows dose- and time-course experiments of mycolactone mediated cytotoxicity on human embryonic lung fibroblasts (HELF). 1A shows best-fit plots and 1B shows raw data plots. **Figure S2** shows mass spectrometry data on mycolactone quantification. Click here for file
