# Supplementary material for: Kinetics of mycolactone in human subcutaneous tissue during antibiotic therapy for Mycobacterium ulcerans disease
Source: BMC Infect Dis. 2014 Apr 15;14:202. doi: 10.1186/1471-2334-14-202 (PMC4021496; doi:10.1186/1471-2334-14-202)
Supplement: Additional file 1 — Experimental data on mycolactone quantification assays. Figure S1A and 1B shows dose- and time-course experiments of mycolactone mediated cytotoxicity on human embryonic lung fibroblasts (HELF). 1A shows best-fit plots and 1B shows raw data plots. Figure S2 shows mass spectrometry data on mycolactone quantification. [file 1471-2334-14-202-S1.pdf]

Additional file 2

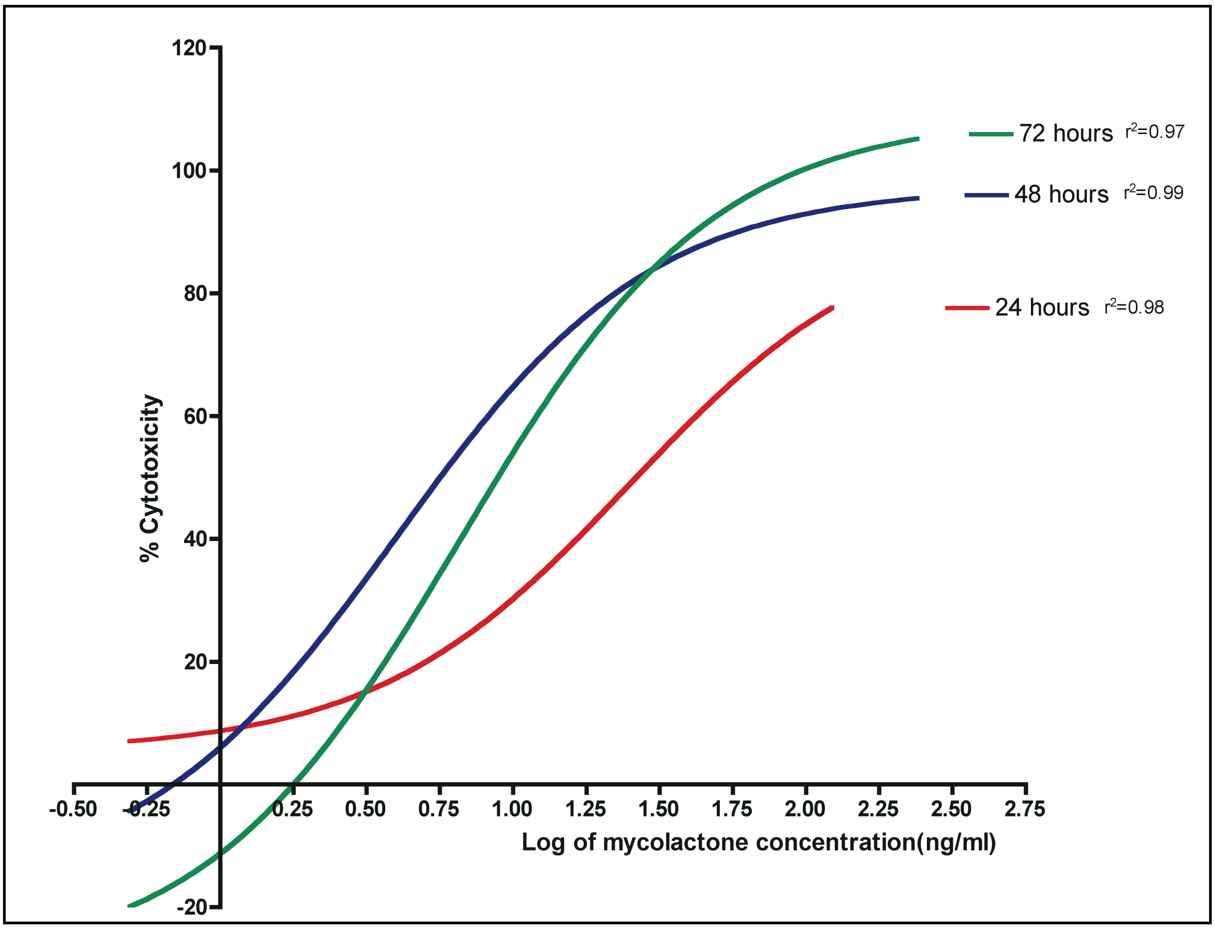

Figure 1A. Best-fit plots of mycolactone mediated cytotoxicity on human foetal lung fibroblasts over 24, 48 and 72 hours measured by MTT assay.

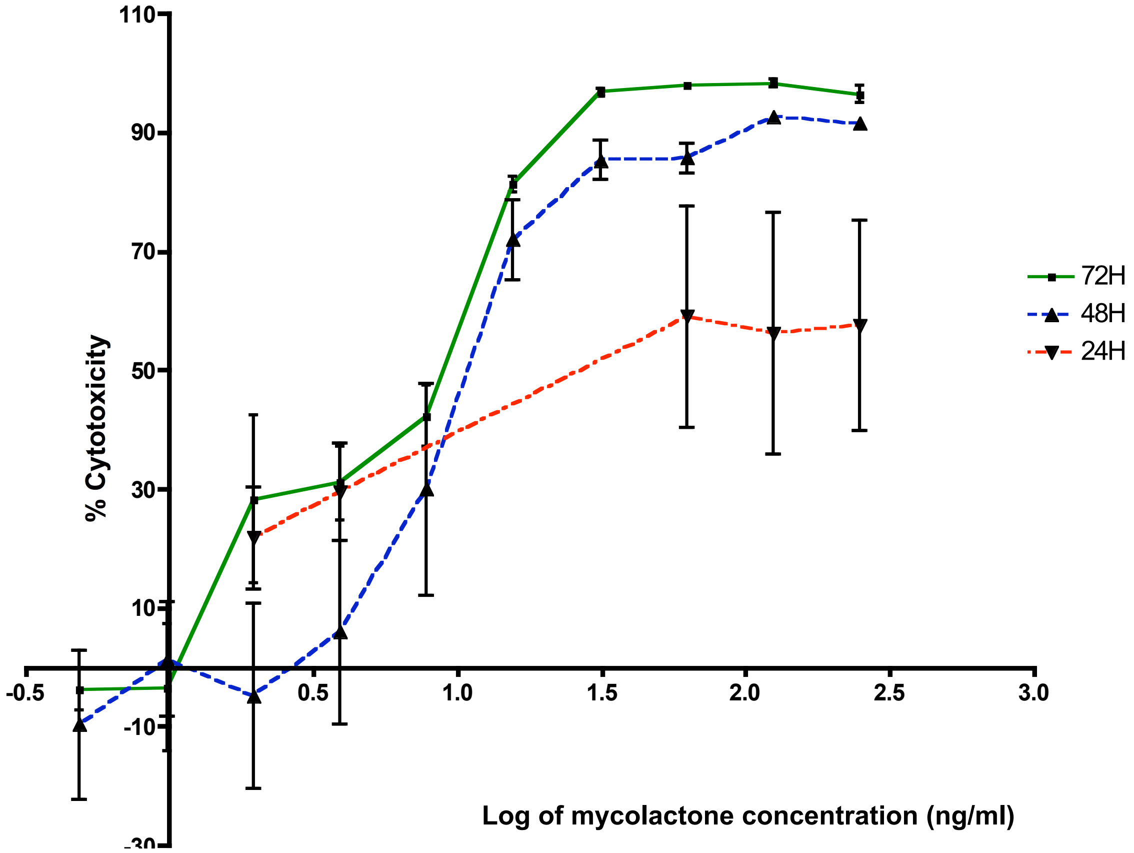

Figure 1B. Raw plots of mycolactone mediated cytotoxicity on human foetal lung fibroblasts over 24, 48 and 72 hours measured by MTT assay. Plots are mean ± SEM or triplicate assays at each concentration.

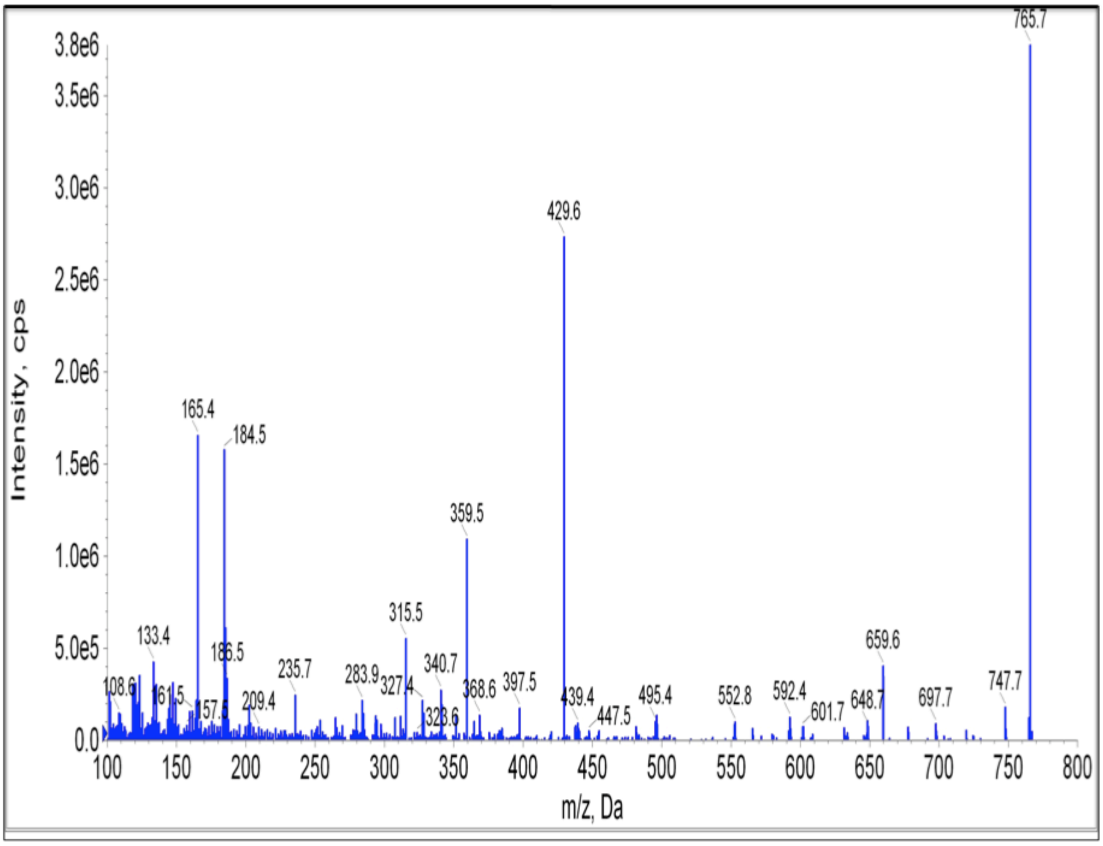
Intensity, cps
m/z, Da
765.7
429.6
165.4
184.5
359.5
315.5
133.4
186.5
659.6
235.7
283.9
340.7
397.5
327.4
368.6
108.6
161.5
209.4
439.4
447.5
495.4
552.8
592.4
601.7
648.7
697.7
747.7

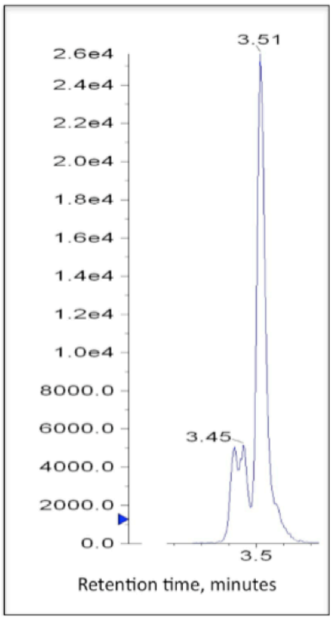
3.51
3.45
Retention time, minutes

Figure 2. Mass spectroscopic analysis using LCQ coupled tandem mass spectrometry for characterisation and quantification of mycolactone A/B; Enhanced Product Ion analysis of the sodiated mycolactone A/B adduct with m/z 765.7, showing core lactone with m/z 429.6 and polyketide side chain with m/z 359.5 from lipid extracts from a representative skin biopsy from an infected human lesion. The extracted ion chromatograms (XIC) for m/z 429.6 which was selected for quantification in LC-MRM analyses of tissue extracts is shown below.
